# Supplementary material for: Importance of consultations using mobile teams in the screening and treatment of neglected tropical skin diseases in Benin
Source: PLoS Negl Trop Dis. 2023 May 12;17(5):e0011314. doi: 10.1371/journal.pntd.0011314 (PMC10208463; doi:10.1371/journal.pntd.0011314)
Supplement: Attachment — Submitted filename: Response to reviewers_2023-02-17.doc [file pntd.0011314.s001.doc]

**Answers to the reviewers**

**Methods section**

**● Reviewer #1**

► **Is mobile consultation the best descriptor to introduce the concept as many will think that this is referring to the use of mobile phone technology to deliver these objectives – see examples in Mali and Botswana for instance.  ? Perhaps  “consultations using mobile teams” would  be better in the title and when first mentioned in the text. If you state this in the title then you can use “mobile teams” in the rest of the manuscript**

***Answer:*** We thank the reviewer for his relevant comment. We agree and propose “consultations using mobile teams”. The term has been modified in the title as well as in the introduction part.

► **The assertion that WHO was encouraging integration for control strategies for NTDs with skin manifestations from 1997 is not accurate – this is a much more recent strategy.**

***Answer :*** Thank you for pointing out this error. The accurate date is 2013. The date has been changed in the article (abstract).

► **Line 128. Please provide figures that show the high levels of endemicity referred to.**

***Answer :*** Dear reviewer, we have modified the sentence and replaced « high levels of endemicity » by « high detection rates » which is more appropriate.

► **It would be useful, for the authors to state clearly the differences between this study and the earlier Benin study – their reference 4. How does it differ – some of the authors are the same ?**

***Answer :*** We can point out several differences between the two studies (the present one and the study concerning reference 4) :

- geographical and social-cultural context were not the same.
- the present study details all the skin diseases identified unlike the previous one
- in the previous study, only 1 campaign was performed in one municipality while here, 3 campaigns were done, in 2 departments
- The two studies were done with two different medical teams in the field.
- In the present study, a dermatologist was present and not in the previous one

However, both studies were supervised by the National Program for the Control of Leprosy and Buruli Ulcer (PNLLUB).

► **What training/preparation was given to the multidisciplinary team. Some were specialists but most were not – so it would be important to show how the teams worked. Did they use written material, diagnostic Apps or field training ?**

***Answer :***

Training was conducted in the field, using materials made available by WHO and the National Program for the Control of Leprosy and Buruli Ulcer (PNLLUB) of Benin.

The trainings focused on the basic epidemiology, the early signs and symptoms of these diseases, some elements of the differential diagnosis, the complications of these diseases when the diagnosis and/or the screening is late, on the modalities of management of these diseases, notably on the existence of a treatment center and the availability of medication.

Power point slides are available if needed.

**● Reviewer #2:**

► **The objectives of the study is not clearly stated and it would be good for the author to clearly state the objectives. Hypothesis they wish to test has also to be clear. The study design seem ok but should be linked to clearly stated objectives.**

***Answer :*** We thank the reviewer for this comment. We have added a sentence in the last paragraph of the introduction part to clearly state the objectives.

**Results section**

**● Reviewer #1:**

► **Can the authors comment on the missing cases ie the patients who were not interested in coming to the clinics – was this a large number ?**

***Answer:*** We had not been confronted with cases of patients who were not interested in our activity. This could be explained by the effectiveness of the sensitization that prevailed before the implementation of this activity, but also by the involvement of the political-administrative and health authorities, especially the involvement of opinion and religious leaders in this activity.

The fact that we did not specify a disease certainly also contributed to the reduction of stigmatization recognized as a factor causing refusals. In fact, our message was addressed to all people with skin lesion, regardless of their origin.

Finally, it is also possible that the fact that the offer was free of charge was a factor in the absence of refusal. In fact, when a patient goes to a health center, he or she pays for the consultation and treatment. In our context, the consultation and treatment were free.

► **Was any feedback provided to the participating communities after the clinics ?**

***Answer :*** This work is part of the activities of the CDTLUB of Pobè which covers this area of the Oueme and Plateau departments. To maintain the link with the communities, a medical team (1 car, 1 doctor, 1 nurse) makes rounds twice a week in this geographical area. Thus, there is a continuous interaction with the community and follow-up of patients.

► **Do you have any more information on the outcomes of the much higher number of cases who had skin diseases which were not NTDs. ie Table 4.**

***Answer:*** All patients diagnosed with a fungal infection received appropriate antifungal treatment which was free of charge. Concerning those diagnosed with a bacterial infection, they received appropriate antibiotic treatment, free of charge.

For other skin pathologies, patients were seen by the dermatologist of the CDTLUB for follow-up and treatment if necessary.

► **The satisfaction of these patients is important to the long term sustainability of this work as it may determine whether people will come forward in future for these mobile skin clinics**

***Answer:*** We have not conducted a formal satisfaction survey but the feedback on these weekly consultations is positive, with the feeling that the population adheres to them. In addition, the center's car continues to visit the villages twice a week and the consultations are always very popular.

We can, however, comment on indirect aspects of this satisfaction. We noticed that the attendance rates of the CDTLUB's health services during and after the mobile consultations increased significantly. The same is true for bed occupancy rates, which were even above 100%. This could be seen as a positive impact of these consultations.

Now, we may have to conduct a community satisfaction survey. Thank you for this remarkable contribution.

► **Table 4 What is epidermophytia ? – how does it differ from ringworm .**

***Answer :*** Ringworm is localized on the head, while epidermophyte is a fungal disease localized on the body.

► **Presumably the mycetoma was bacterial or was it fungal in which case it should be with the fungal infections**

***Answer :*** the case of mycetoma was bacterial. Diagnosis was confirmed by anatomical pathology.

► **What is fibrosing folliculitis – is this scarring alopecia in which case should it be tumoral, or is it acne keloid ?**

***Answer :*** fibrosing folliculitis is consecutive to a *Staphylococcus aureus* folliculitis. This is to be distinguished from acne keloid, but also from post-cicatricial keloids, consecutive to skin wounds and/or trauma.

► **Hot abscess – abscess or furunculosis ?**

***Answer :*** The term "hot abscess" is used in clinical practice and on the semiological level. It is distinguished from “cold abscess” whose etiology refers to a specific germ such as Mycobacterium. Hot abscess is therefore used to say that it is an abscess that has the cardinal signs of inflammation which is characterized by redness, heat, pain, swelling. It is often unique, localized on a part of the body and in our context; it is often of staphylococcal origin.

► **Herpes – presumably Herpes simplex**

***Answer :*** Yes it is Herpes simplex. The term “simplex” has been added in table 4

► **Sudamina – miliaria**

***Answer :*** both terms can be used. We have added “miliaria”, in brackets

**● Reviewer #2:**

►**The analysis is fine and results clearly presented. Images can be improved - quality images.**

***Answer :*** quality of images has been improved (tiff. Format). Images in tiff format have been added in the new submission.

**Conclusions section**

**● Reviewer #1:**

► **From line 449 onwards there are a series of actions listed that would help to sustain this work – how many of these have been implemented ?**

***Answer:*** Weekly visits continue to be carried out and consultations using mobile teams are planned in 2023 in districts not yet covered by the mobile team.

Moreover, Dr Gnimavo (first author of the article), who was working at the CDTLUB and supervised the mobile consultations has recently changed position. He became the head doctor of the district in the same commune. He now coordinates all public health activities, including patient follow-up, in both covered and uncovered areas. This is an advantage for strengthening these field activities.

► **What impact has COVID 19 had on this project ?**

***Answer:*** 2018 and 2019 were not concerned by COVID and the project has been little impacted in 2020. The mobile team continued its activities during which the barrier measures were strictly respected. The ministry authorized the consultations with some requirements. Thus, all the members of the team used kits containing masks and gloves. They also used hydro-alcoholic solutions

.

**● Reviewer #2:**

► Discussions be limited to the data/results. Extrapolations not supported by the data are not needed. Extensive discussions of intersectoral collaboration are not needed. It is easy to say but difficult to do.

***Answer:*** We thank the author for his contribution to the improvement of this work. We do not extrapolate but we are in a prospective vision and a holistic approach to solve the health problems raised by our study.

We do not wish to limit ourselves to comparing the results of our study to those of other authors and to present the limits of our study. We must propose relevant solutions capable of circumscribing the observed public health phenomenon.

In our study, we have highlighted an epidemiology dominated by infectious dermatoses with scabiosis in particular. We think it is appropriate to propose actions that could put an end to this public health problem. The proposed solutions may be difficult but not impossible.

It seems clear to us that if we continue to organize active screenings in the community without integrating the health promotion aspects, in 10, 15, or 20 years, the health problems we have observed today will continue and even get worse. We must therefore not remain solely at the level of integration of diseases, but we must go to the integration of control strategies taking into account intersectoral collaboration. This is the vision advocated by the new WHO strategic document for the year 2030. Finally, we must not hide the fact that this manuscript is also a public health document.

► The strategy of mobile outreach has been used for decades but to see it used in Benin in the context of skin NTDs surveillance and diagnosis has provided good data on prevalent skin diseases in the surveyed areas. Large number of skin diseases are not skin NTDs and their management challenges should be discussed in detail.

***Answer:*** All patients diagnosed with a fungal infection received appropriate antifungal treatment which was free of charge. Concerning those diagnosed with a bacterial infection, they received appropriate antibiotic treatment, free of charge.

For other skin pathologies, patients were redirected to a dermatologist of the CDTLUB for follow-up and treatment if necessary.

**Editorial and Data Presentation Modifications**

**● Reviewer #1:**

►**Please check spelling and references as there are some errors**

***Answer :*** reference 15 has been replaced and reference 42 has been corrected.

**● Reviewer #2:** (No Response)

**Summary and General Comments**

**● Reviewer #1:**

►**Overall this is interesting work. It would be good to hear more about how the authors intend to ensure sustainability of this work.**

***Answer:*** Weekly visits continue to be carried out and consultations using mobile teams are planned in 2023 in districts not yet covered by the mobile team.

Moreover, Dr Gnimavo (first author of the article), who was working at the CDTLUB and supervised the mobile consultations has recently changed position. He became the head doctor of the district in the same commune. He now coordinates all public health activities, including patient follow-up, in both covered and uncovered areas. This is an advantage for strengthening these field activities.

**● Reviewer #2:**

►**Please use Skin NTDs rather than cutaneous NTDs**

***Answer:*** the term “cutaneous NTDs” has been replaced by “skin NTDs” in the whole article

►**Change cutaneous to skin**

***Answer:*** the term “cutaneous” has been replaced by “skin” in the whole article

**►Get a native English speaker to review the paper**

***Answer:*** the article has been reviewed by a native english speaker

**►Check reference and use the most recent references to NTDs.**

***Answer:*** references have been checked. Reference 15 has been replaced.

**►Line 111: Please check the statement on eradication of the three diseases carefully. Please refer to the NTD Road Map 2021-2030. Buruli ulcer is for control, leprosy for elimination and yaws for eradication.**

***Answer:*** modifications have been done in the text : “... in which one of the targets identified was the elimination of leprosy, control of Buruli ulcer and eradication of yaws”.

**►Line 179: individuals with dermatological lesions not regions**

***Answer:*** “regions” has been replaced by “lesions”

**►Line 195: Yaws and not yaw**

***Answer:*** “yaw” has been replaced by “yaws”

**►Line 194: Please provide reference or revise the sentence.**

***Answer:*** the reference (ref no. 20) has been added

**►Line 197: BU swabs are taken from the undermined edges of the ulcer (please make this clear)**

***Answer:*** “taken from the undermined edges of the ulcer” has been added for greater clarity

**►Line 277: Use male or female instead of man and woman**

***Answer:*** “man” has been replaced by “male” and “woman” by “female”

**►Line 281: Use debridement instead of lesion trimming**

***Answer:*** “trimming” has been replaced by “debridement”

**►Line 285: What multidisciplinary team was in the community?**

***Answer:*** In the community, the multidisciplinary team was composed of a health agent of the health area, local elected officials, community relays, village leaders, town criers, religious and opinion leaders as well as local teachers.

**►Line 361: How did the strategy helped to reduce the burden of these diseases?**

***Answer:*** One of the objectives of the mobile clinics is to ensure regular screening as early as possible. Thus, treatment can be initiated quickly in positive patients. In addition, contact between positive patients and people is limited and thus transmission of leprosy is reduced.

**►Line 480-493: Discussions outside the data generated. Kindly discuss the results and avoid extrapolation to what is not provided by data collected. Inter-sectorial collaboration is good but very hard to achieve.**

***Answer:*** explanations were provided above : « We do not extrapolate but we are in a prospective vision and a holistic approach to solve the health problems raised by our study.

We do not wish to limit ourselves to comparing the results of our study to those of other authors and to present the limits of our study. We must propose relevant solutions capable of circumscribing the observed public health phenomenon.

In our study, we have highlighted an epidemiology dominated by infectious dermatoses with scabiosis in particular. We think it is appropriate to propose actions that could put an end to this public health problem. The proposed solutions may be difficult but not impossible.

It seems clear to us that if we continue to organize active screenings in the community without integrating the health promotion aspects, in 10, 15, or 20 years, the health problems we have observed today will continue and even get worse. We must therefore not remain solely at the level of integration of diseases, but we must go to the integration of control strategies taking into account intersectoral collaboration. This is the vision advocated by the new WHO strategic document for the year 2030. Finally, we must not hide the fact that this manuscript is also a public health document”.
